# Postoperative thoracogastric necrosis associated with thoracogastric–tracheal fistula of an endoscopic McKeown-type resection of esophageal carcinoma: A case report

**DOI:** 10.1097/MD.0000000000028755

**Published:** 2022-02-04

**Authors:** Yanhong Lu, Zixue Ren

**Affiliations:** Department of Thoracic Surgery, The First Affiliated Hospital of University of Science and Technology of China (Anhui Provincial Cancer Hospital), Hefei, China.

**Keywords:** case report, thoracogastric necrosis, thoracogastric tracheal fistula

## Abstract

**Introduction:**

Postoperative thoracogastric necrosis (TGN) associated with thoracogastric-tracheal fistula (TGTF) of an endoscopic McKeown-type resection of esophageal carcinoma is rare and has a poor prognosis and high mortality. Few cases have been reported and successful treatment is rare. Surgery is the major treatment option.

**Patient concerns:**

A 71-year-old man was hospitalized in a local hospital for more than 2 months due to dysphagia. The patient was previously healthy and had no underlying diseases.

**Diagnosis:**

TGN associated with TGTF of an endoscopic McKeown-type resection of esophageal carcinoma.

**Intervention:**

Two-stage surgeries were performed.

**Outcome:**

The patient recovered well at the time of the follow-up examination on April 4, 2021 with an ECOG score of 0.

**Conclusion:**

Staging surgery can be an alternative treatment for TGN associated with TGTF of an endoscopic McKeown-type resection of esophageal carcinoma.

## 1 Introduction

Thoracogastric necrosis (TGN) associated with thoracogastric-tracheal fistula (TGTF) after esophageal cancer is a potentially fatal complication.^[1]^ The incidence of TGN associated with TGTFs after esophageal cancer is very rare. The most common symptom of TGN associated with TGTF after esophagectomy^[1,2]^ is cough after swallowing, and the nasogastric tube (NGT) cannot produce a negative pressure. Continuous inhalation of digestive juices contaminates the respiratory system, which can lead to breathing difficulties and, eventually, respiratory failure. Despite the severity of TGN associated with TGTFs, no ideal treatment is recommended. I have shared a case that was successfully cured by staged surgery.

## 2 Case presentation

A 71-year-old man was admitted to a local hospital for more than 2 months due to dysphagia. Gastroscopic pathological biopsy revealed esophageal squamous cell carcinoma G2 located 32 to 36 cm from the medial incisor. The patient visited our hospital for further treatment.

**Figure 1:**
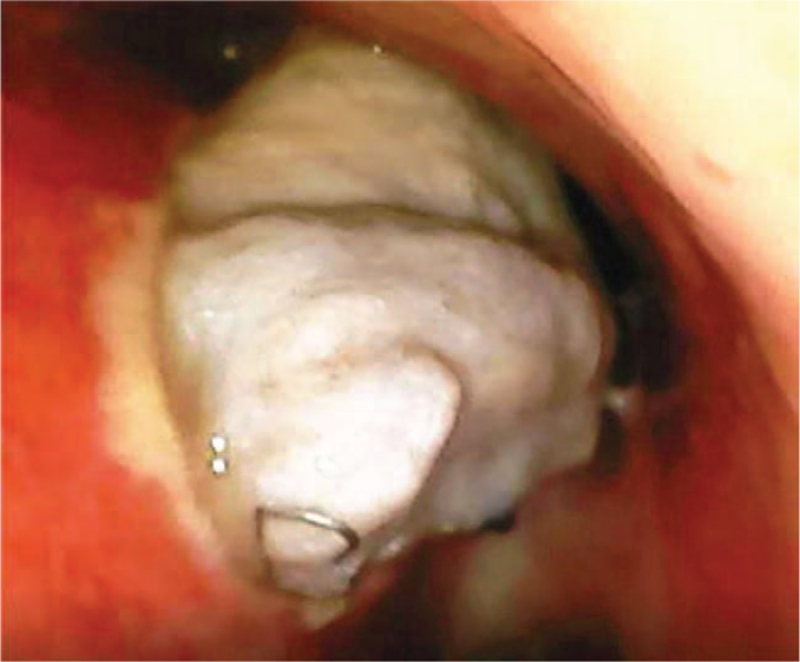
The trachea fistula was about 0.5 cm above the tracheal carina.

**Figure 2:**
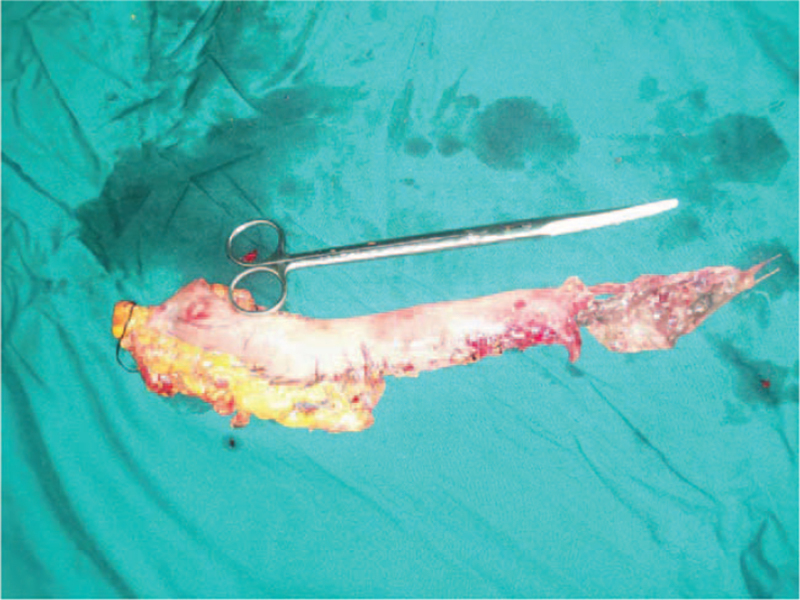
The thoracogastric necrosis was at the level of azygos vein bow to anastomosis.

The patient was previously healthy without any underlying diseases and underwent a more advanced evaluation that revealed no obvious contraindications to surgery. PET-CT revealed that the clinical stage was cT2-3N0M0 IIA-IIB. An endoscopic McKeown-type resection of the esophageal carcinoma was performed on December 18, 2019. During postoperative pathology, an esophageal squamous cell carcinoma, G2, lymph node (0/41), pT3N0M0, stage IIB, was discovered. On December 24, 2019 the right thoracic drainage tube was drained with approximately 1100 mL of a pale yellow, purulent, odorous liquid, which was considered to have been caused by an anastomotic fistula. Due to an aggravated pulmonary infection, on December 29, 2019, the patient was transferred to the ICU and underwent bronchoscopy (Fig. 1), which indicated a thoracogastric–tracheal fistula. On December 30, 2019 thoracogastric necrosis was observed during surgical treatment (Fig. 2). Necrosis extending from the azygos vein bow to the anastomosis. The fistula was approximately 0.5 cm above the tracheal carina and had a diameter of 2 cm. The removal of a pedicled pericardium (dimensions: 5 cm × 4 cm × 3 cm) and transplantation of the omentum were performed to repair and close the tracheal fistula. Esophagostomy, tubular gastrectomy, and jejunostomy were performed.

Although the patient recovered smoothly and was discharged from the hospital, several surgical issues were worth sharing in the second operation: Anesthetic factors: The anesthetist intubated the trachea and closed the lungs. During chest surgery, the tracheal fistula enlarged, and serious air leakage occurred. The operation was halted and the anesthetist replaced the endotracheal tube with a double-lumen endotracheal tube. Surgical factors: During the operation, the thorax was found to be full of purulent secretions and rinsed with distilled water. Although double-lumen endotracheal intubation blocked the left main bronchus, a large amount of water soon entered the bronchus. Hydrops in the thorax and water entering the left lung were rapidly suctioned. Therefore, the patient's vital signs were stable.

Nonetheless, the operation was successful in several important aspects that aided the patient's recovery. During the second surgery, the greater omentum was used for reinforcement after pedicled pericardial repair, which reduced the possibility of a recurrent tracheal fistula by acting as a second line of defense. Thoracogastric resection was performed during the second operation because of thoracogastric necrosis. However, due to severe chest infection, colon replacement esophagostomy was not performed during the second surgery after the fistula was repaired. For patients undergoing pericardial repair of a tracheal fistula, the first-line treatment is esophageal colon surgery. However, any preexisting pleural infection is likely to be more severe after the operation, which would progressively weaken the patient. The vast majority of colon esophageal anastomotic fistulas occur at this stage and carry the risk of heart damage in the case of a damaged pericardium, thus raising the possibility of death. During the second surgery, the cervical esophageal stump was stitched and fixed together with the neck drainage tube. In the third operation, the original neck adhesions were severe. The esophagus was not detected until the surgeon made an incision along the fixed neck drainage tube.

**Figure 3:**
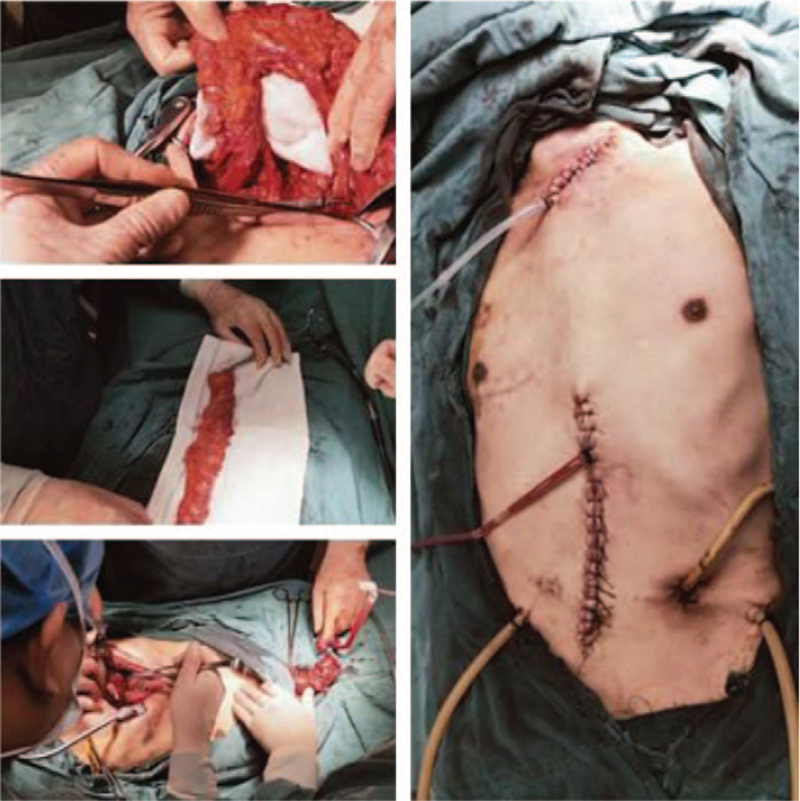
Colon replacement esophagostomy under the subcutaneous tunnel of sternum + colonic colonic anastomosis + jejunal colonic anastomosis.

The procedure was completed in a smooth manner. The patient recovered successfully and was discharged on February 22, 2020. After the surgery, the patient received enteral nutrition via the nutrient canal. ECOG score:1. On July 3, 2020 a third operation (colon replacement esophagostomy via a subcutaneous tunnel over the sternum, colon–colon anastomosis, and jejunum–colon anastomosis) was performed at our hospital (Fig. 3). During the third operation, colon replacement esophagostomy through the subcutaneous tunnel of the sternum was selected instead of a thoracic approach, which reduced the damage to cardiopulmonary function, promoted the success of the operation, and accelerated postoperative recovery.

The patient was discharged in healthy condition on July 20, 2020. He had recovered well by the time of the follow-up examination on April 4, 2021 with an ECOG score of 0.

## 3 Discussion

Postoperative thoracogastric necrosis is a rare and serious complication of esophageal surgery.^[1]^ The patient presented with thoracogastric necrosis above the azygos arch level, which was thought to have been caused by the following^[1]^: Excessive traction was applied when the thoracogastric tube was anastomosed to the neck. The elderly patient suffered from chronic atrophic gastritis, which weakened the gastric wall, and the vascular network in the gastric wall was damaged due to traction.

The following may have been a potential cause of thoracogastric–tracheal fistula in this patient^[2–5]^: Thoracogastric factors Thoracogastric necrosis may have caused an anastomotic fistula and enabled a large amount of gastric acid to enter the thorax and corrode the trachea. Surgical factors: During intraoperative dissection of the subcarinal lymph node, high-energy instruments such as ultrasonic and electric scalpels may accidentally injure the bronchial membrane. Anesthetic factors: The endotracheal intubation balloon may have been overinflated, or excessively deep double-lumen endotracheal intubation may have damaged the bronchial membrane.

Because of the close relationship between the thoracogastric tube and tracheal membranes, necrotic perforation of the thoracogastric tube and anastomosis with the trachea lead to a thoracogastric–tracheal fistula. Thoracogastric necrosis associated with thoracogastric-tracheal fistula was found in an earlier case, and the patient was able to tolerate surgery, indicating that surgical treatment was undoubtedly the best treatment plan.

The main clinical manifestations of thoracogastric-tracheal fistula and thoracogastric necrosis are a severe irritating cough, gastric fluid-like sputum, and severe pulmonary infection.^[2]^ Instead of blindly fighting the infection and treating the cough, an imaging examination should be performed in a timely manner so as not to delay the diagnosis and cause serious irreversible consequences such as respiratory failure and organ failure. In the present case, a large amount of gas was continuously discharged from the gastrointestinal decompression tube after the operation, with obvious moist rales in the lungs, progressive aggravation, and a severe irritating cough. Therefore, we considered a thoracogastric tracheal fistula, which was confirmed by tracheal examination. In a clinical examination, a potential thoracogastric–tracheal fistula cannot be ruled out, even if gastroscopy suggests no signs of fistula.^[2]^ Gastric fold can cause the false appearance of occlusion of the fistula; therefore, the possibility of a thoracogastric–tracheal fistula should be excluded by bronchoscopic analysis.

TGN combined with TGTF after esophageal cancer is rare, and reports in the literature are mainly related to TGTF. Therefore, no ideal treatment is recommended. General conservative treatments include nutritional support and stenting. However, Boyd^[6]^ suggested that airway stenting should not be considered as a final treatment because of its high recurrence rate (39%). Endoscopic injection of fibrin glue^[7]^ and a new endoscopic clamp device^[8]^ can also effectively close the TGTF. A pedicled subcutaneous fascia flap wrapping the trachea has been reported in the literature for TGTF,^[2]^ but the mortality rate is higher, up to 64.3%. Sato^[9]^ reported that the use of a 3-step surgery could be an effective procedure for TGTF. Above all, there have been no reports on the successful treatment of patients with TGN associated with TGTF during staged esophageal surgery. Therefore, a staged operation can be used as a surgical option.

## Author contributions

**Data curation:** Yanhong Lu.

**Formal analysis:** Yanhong Lu.

**Investigation:** Yanhong Lu.

**Project administration:** Yanhong Lu.

**Supervision:** Yanhong Lu, Zixue Ren.

**Writing – original draft:** Yanhong Lu.

**Writing – review & editing:** Yanhong Lu.
